# Undergraduate nursing students’ self-reported professional behaviour at the University of Namibia

**DOI:** 10.4102/hsag.v26i0.1703

**Published:** 2021-11-30

**Authors:** Nestor Tomas, Alpheus K. Ndjamba, Takaedza Munangatire

**Affiliations:** 1 Department of General Nursing Science, Faculty of Health Science, University of Namibia, Rundu, Namibia

**Keywords:** undergraduate, nursing student, professional behaviour, self-reported, caring

## Abstract

**Background:**

Development of professional behaviour in nursing students is an important part of a nurse’s overall competence. Self-evaluation is one way of measuring professional behaviour amongst nursing students. However, studies on self-reported professional behaviour of nursing students are limited in Namibia.

**Aim:**

This study aimed to investigate nursing students’ self-reported professional behaviour at the University of Namibia.

**Setting:**

The setting was a university campus offering a Bachelor of Nursing Science degree in Namibia.

**Methods:**

A quantitative descriptive contextual design was used with 100 nursing students. Data were analysed descriptively using a non-parametric Kruskal–Wallis and ANOVA tests of variance and statistical significance.

**Results:**

High mean scores were found in the areas of utilising evidence-based solutions (4.78 ± 0.58), promoting clinical teaching (4.46 ± 0.94), willingness to implement quality improvement initiatives (4.34 ± 0.518), and protecting health, safety and patient’s rights (4.28 ± 0.55). The lowest mean scores were recorded in projecting professional image (2.22 ± 1.27), rendering evidence-based care (4.08 ± 0.44). The study found statistical significance difference between self-reported professional competency (*p* = 0.01) and quality care improvements (*p* = 0.02).

**Conclusion:**

In this study, nursing students’ self-reported professional behaviour was rated high (mean scores > 4.0 out of 5). Despite this high rating, it cannot be concluded that the students were professionally competent. We recommend that professional behaviour be measured from both students’ and nurse educators’ or patients’ perspectives.

**Contribution:**

The findings from this study provide supplementary evidence on self-reported professional behaviour with implications on nursing education and practice.

## Introduction

Nursing students are expected to develop competence and this includes good professional behaviour, nursing values and ethics (Riklikiene, Karosas & Kaseliene 2018:1). Professional nursing values such as trustworthiness, impartiality, honesty and objectivity should be an integral part of the nursing students’ development (Haahr et al. 2020:1). The development of nursing students’ professional behaviour should be attained through the process of socialisation with nursing educators (Magopeni 2013:2; Poorchangizi et al. (2019b:2). Nursing students should also be able to self-evaluate their development including their professional behaviour. However, studies on self-reported professional behaviour are limited despite the significance of good professional behaviour in determining the nursing students’ competence. Therefore, this study sought to assess professional behaviour from nursing students’ perspective.

Professional behaviour is an important component of nursing competence. The components of professional behaviour which include ethics, caring attitudes, honesty, respect, communication, time management, integrity, dressing, and accountability play a significant role in determining the quality of nursing care rendered (Charania et al. 2017:1). In addition professional behaviour is associated with some form of etiquette in the workplace that results in respectful conduct (Kowalski 2016:1). Such respectful conduct that comes with professional behaviour makes one conscious of how to react to co-workers and clients, hence generating a positive nursing care environment (Karimi et al. 2014:10). Consequently, it is necessary to explore nursing students’ professional behaviour because competence in professional behaviour is likely to improve nursing care.

Development of professional behaviour is marked by three dimensions: cognitive, attitudinal and psychomotor (Ghadirian, Salsali & Cheraghi 2014:8). Cognitive dimension deals with an understanding of the underlying principles of professional conduct that a student must prioritise, attitudinal dimension deals with the professional values that must shape the behaviour, whereas psychomotor dimension includes the acceptance and implementation of professional values. In the process of professional development of professional behaviour, various social, political, cultural, scientific and technological factors should be considered. (Dikmen et al. 2016:1). Also for students to develop professional behaviour, they need to display enthusiasm in the values and behavioural attributes of nursing (Tanaka et al. 2017:2). It is therefore necessary to assess and monitor development of professional behaviour in nursing students.

Both health industries and communities expect that nursing graduates pass stringent tests to prove that they are responsible, accountable and demonstrate good professional behaviour (Magopeni 2013:4). Similarly, nursing regulators put the responsibility upon nurses to engage in professional behaviour, preserve professionalism and the reputation of nursing (Republic of Namibia 2004:s9; Shohani & Zamanzadeh 2017:2). Despite having signed codes of conduct, nursing students sometimes engage in unprofessional behaviour. Such unprofessional behaviour can be attributed to absence of good role models resulting in negative effects on patients, the student’s career and overall professional image (Matshabane 2016:1; Sealy & Singh 2008:2). The early identification of student behaviour is necessary to ensure they obtain adequate support and guidance in order to have good professional behaviour (Poorchangizi et al. 2019a:1). Assessing students’ self-reported professional behaviour can aid the faculty to know the level of professional behaviour which is important in crafting more effective codes of conduct and educational policies (McClung & Schneider 2018:1).

In Namibia, nursing is regulated by the Nursing Council of Namibia (NC) established by an act of parliament *Nursing Act No. 8*, 2004 with the objective to protect the public through regulated education and practice of nurses (Republic of Namibia 2004:9). Nursing students register with the NC at the beginning of their education and undergo education and training on professionalism amongst other key components of nursing education. In line with the Ministry of Health and Social Services’ mission, the mission of the University of Namibia (UNAM), School of Nursing, is to facilitate development of professionalism amongst nursing students (UNAM 2019:viii). Notwithstanding the focus of School of Nursing on professional behaviour, the researchers observed that a number of nursing students failed to uphold good professional behaviour even at the point of graduation. Moreover, there has been an outcry from the public regarding the unprofessional behaviour of nurses (Tomas, Maboe & Mamahlodi 2019:113). Failure to monitor and assess nursing students’ professional behaviour may result in nursing education producing graduates who lack professionalism. For that reason, this study assessed the nursing students’ (second, third and fourth years) self-reported professional behaviour at UNAM.

### Research objectives

The objective of this study was to assess and describe nursing students’ self-reported professional behaviour at UNAM.

## Methods

### Research setting

This study was conducted at the UNAM satellite campus, in the Eastern part of Namibia. The satellite campus has an enrolment of about 4000 students. Courses offered at the campus include degree in Nursing Science, Education and Economics and Management Sciences.

### Research design

A quantitative, descriptive and contextual approach was used to describe and measure the nursing students’ self-reported professional behaviour. Descriptive technique enabled the researchers to reach a large number of nursing students and draw required conclusion about the variables (Brink, Van der Walt & Van Rensburg 2012:97).

### Population and sampling strategy

The population for this study included a total of 250 undergraduate nursing students from second to fourth year of study, enrolled for the Bachelor of Nursing Science (clinical degree) at UNAM. As cited in Abimana, Kato and Bazira (2019:2), this study used Solvin’s formula for estimating sample size, $\frac{N}{1+N\times a^{2}}$ to estimate the population sample with ‘*N*’ being the total population and ‘*a*’ being the confidence limit at 95%, with 0.05 error margin. A total of 154 respondents from the second to fourth year levels were selected for this study. Respondents in this study were nursing students who had a year or more of clinical exposure and were expected to be well rounded with the expected standard of ethics of the nursing profession. Recruitment of respondents was performed in November 2020 via academic WhatsApp groups, using a non-probability convenient sampling technique. This approach allowed for the selection of conveniently available respondents with data bundles and those who were available during the period of data collection. The researchers extended data collection period to 4 weeks to allow all eligible respondents to participate. Online questionnaires were preferred in order to adhere to coronavirus disease 2019 (COVID-19) social distance regulations.

### Data collection and instrumentation

Data were collected using a self-administered online questionnaire. The questionnaire had two sections, the first of which asked for sociodemographic variables whilst the second section included questions that measured the students’ self-reported professional behaviour, using 14 items on a 5-point Likert scale, rated from 1 = never to 5 = always. The scores of the responses were aggregated to a maximum of 70 scores for self-reported professional behaviour. The mean scores were then classified and interpreted as follow: 4–5 = good, 2.6–3.9 = satisfactory and 1.0–2.5 = poor professional behaviour.

The researchers developed the questionnaire using literature related to professional behaviour in nursing. Some aspects measuring self-reported professional behaviour in the context of Namibia were extracted from the existing behaviour inventory tools developed by Van Mook (2011:109) and May, Kontney and Iglarsh (2010:2).

Three nurse education specialists checked the questionnaire items for clarity and alignment with the research objectives. The instrument was pre-tested on eight students using Google Forms®. Following the checking and pretesting of the instrument, corrections were made to improve the validity of the instrument. Reliability was ensured by administering the validated questionnaire twice with a 2 week gap between the first and the second administration to a group of nursing students. Cronbach’s alpha coefficient test was performed and the alpha value was α = 0.71, greater than the acceptable level of α = 0.70.

Explanatory factor analysis (EFA) was further performed to validate the validity of the data collection tool. Watkins (2018:5) found EFA an acceptable validity test for instruments with closed-ended questions, such as the Likert scale questions. The results showed that the Kaiser-Meyer-Olkin sampling adequacy score was 0.79, greater than 0.70, as outlined by Taber (2018:1). The final results of the study only considered those items that reported Eigenvalues > 1 on the EFA.

Data for the main study were collected for 2 weeks, from 23 November 2020 to 05 December 2020. An online questionnaire (Google Forms) was used to collect data because of COVID-19 restrictions on gatherings. The online questionnaire had two sections, the one with information and consent for respondents and the other with the questions. A link was shared through academic WhatsApp groups with an introductory message. Interested respondents who clicked on the link would first access the electronic informed consent. As the next step, they were required to click ‘the agree button’ to give consent for participation before proceeding to complete the questionnaire. All responses were automatically populated and recorded on Google Forms®. No identifying data were collected to ensure anonymity and confidentiality. Although respondents were permitted to withdraw from the study anytime without any punitive measure, none of the respondents withdrew from the study.

### Data analysis

Data were analysed using Statistical Package for the Social Sciences (SPSS) version 26.0. Descriptive analysis was applied for demographic variables. Mean and standard deviation (SD) were reported to describe the scores of the self-reported professional behaviour. The relationship between self-reported professional behaviour and demographic variables were analysed using one way- analysis of variance (ANOVA) for normally distributed data. For data that were not normally distributed the non-parametric Kruskal–Wallis test was applied to compare differences of the mean scores between groups.

### Ethical considerations

The study was conducted according to the Declaration of Helsinki (World Medical Association 2001:1) observing all ethical principles. The study was granted approval from the School of Nursing Ethical Committee of the UNAM, reference number: SoNEC 89/2020. Every participant was provided with written information about the purpose of the study by the researchers and informed consent was obtained from all the participants. Participants were further informed that their participation was voluntary and that they could withdraw at any time without any prejudice. Confidentiality and anonymity were ensured through the use of a non-identifiable electronic questionnaire.

## Results

### Demographic data of the respondents

Of the 154 online questionnaires, only 100 were completed and returned, giving a response rate of 65% (*n* = 100). A total of 73% (*n* = 73) of the respondents were aged between 21 and 30 years, 22% (*n* = 22) between 16–20 years and 5% (*n* = 5) were aged 31 years and above. The mean age was 26 years and the SD was 5.00. Amongst the respondents, 57% (*n* = 57) were females and 43% (*n* = 43) were males. In terms of the year of study, both the third year and the second year accounted for 33% (*n* = 33) of the respondents respectively, and 34% (*n* = 34) were in the fourth year. Almost all respondents, 96% (*n* = 96) were Christians, whilst 4% (*n* = 4) represented other religions.

Table 1 shows the distribution of the professional behaviour mean scores. Across the 15 subscales, the scores were high in the following areas: utilising evidence-based solutions in decision making (4.78 ± 0.58), promoting clinical teaching (4.46 ± 0.94), willingness to work on quality improvement initiatives (4.34 ± 0.51), practice with compassion and respect for every individual (4.31 ± 0.56), protect the health, safety and rights of the patients (4.28 ± 0.55), and advancing the profession through practice education and knowledge (4.25 ± 0.52). The low mean scores were recorded in the following areas: projecting a professional image (2.22 ± 1.27), rendering evidence-based care (4.08 ± 0.44), and patient advocacy (4.09 ± 0.51).

**TABLE 1:** Distribution of subscale mean scores pertaining to self-reported professional behaviour.

Variable	Mean	Standard Deviation	Minimum	Maximum	Overall score Mean ± SD
Collaborate with other healthcare professionals and community	4.16	0.42	3	5	4.11 ± 0.57
Advocate for patients	4.09	0.51	3	5	
Advance the profession through practice, education and knowledge	4.25	0.52	3	5	
Initiate actions to improve daily practice	4.12	0.48	3	5	
Protect the health, safety and rights of the patient	4.28	0.55	3	5	
Project professional image	2.22	1.27	1	5	
Render evidence-based patient care	4.08	0.44	3	5	
Uses a cell phone while on duty	4.12	1.02	1	5	
Seek and implement solutions	4.13	0.68	2	5	
Promote clinical teaching	4.46	0.94	1	5	
Utilise evidence-based solutions in decision making	4.78	0.58	2	5	
Maintain appropriate relationships with patients, fellow students and lecturers/seniors	4.18	0.46	3	5	
Practice with compassion and respect for every individual	4.31	0.56	3	5	
Willingness to work on quality improvement initiatives	4.34	0.51	3	5	

SD, standard deviation.

Table 2 shows the association of demographic data (age, gender, religion and year of study) to the self-reported professional behaviour scores. The study reported a higher overall mean score (4.11 ± 0.57) for the self-reported professional behaviour. Of the elements measured, self- reported professionalism received the highest mean score in the age group of 16–20 years (4.71 ± 0.71), in females (4.56 ± 0.91), amongst Christians (4.53 ± 0.87) and in the fourth year students (4.65 ± 0.91).

**TABLE 2:** Association between demographic characteristics and self-reported professional behaviour (*n* = 100).

Variable	Demographic	Characteristics	Mean	Standard deviation	*p* *
Quality care improvements	Age (in years)	16–20	4.10	0.30	0.74
		21–30	4.16	0.43	
		31 and above	4.20	0.44	
	Gender	Male	4.20	0.45	0.34
		Female	4.12	0.37	
	Religion	Christian	4.13	0.39	0.02
		Others	4.67	0.57	
	Year of study	Second year	4.21	0.48	0.26
		Third year	4.18	0.39	
		Fourth year	4.06	0.34	
Self-reported professional competency	Age (in years)	16–20	4.00	0.44	0.59
		21–30	4.08	0.43	
		31 and above	4.20	0.44	
	Gender	Male	4.10	0.43	0.59
		Female	4.05	0.43	
	Religion	Christian	4.05	0.41	0.01
		Others	4.67	0.57	
	Year of study	Second year	4.09	0.45	0.80
		Third year	4.03	0.39	
		Fourth year	4.09	0.45	
Self-reported professionalism	Gender	Male	4.44	0.89	0.23
		Female	4.56	0.91	
	Age (in years)	16–20	4.71	0.71	0.45
		21–30	4.46	0.92	
		31 and above	4.40	1.34	
	Religion	Christian	4.53	0.87	0.65
		Others	4.00	1.73	
	Year of study	Second year	4.42	0.93	0.15
		Third year	4.45	0.86	
		Fourth year	4.65	0.91	

* , Kruskal–Wallis test.

Self-reported professional competency received a higher mean score in respondents of the 30 years and above (4.20 ± 0.44) and in other religion category (4.67 ± 0.57). The lowest mean score of self-reported professional competency was recorded amongst younger respondents in the 16–20 years age category (4.00 ± 0.44) and amongst the females (4.05 ± 0.43). With regard to quality care improvement, other religion scored the highest mean score (4.67 ± 0.57), for male category (4.20 ± 0.459) and in the age category of 31 years and above (4.20 ± 0.44). The lowest mean score of quality care improvement was recorded amongst fourth year students (4.06 ± 0.34).

The study found a strong association between religion and self-reported professional competency (*p* = 0.01) and religion and quality care improvements (*p* = 0.02). No statistical significance was found between ages, gender and level of study with the study variables (*p* ≥ 0.05) (see Table 2).

## Discussion of the results

### Demographic characteristics

The study showed that majority of the respondents 73% (*n* = 73) were in the age group of 21–30 years, showing that the majority of undergraduate nursing students are young. This pattern concurs with a previous study conducted between 2002 and 2009, which indicated that more people become nurses in their 20s (Beurhaus 2011:7). However, researchers predicted that although young nurses enter the profession, challenges related to ageing nursing workforce remain a reality in many countries (Sherman, Chiang-Hanisko & Koszalinski 2013:1; Uthaman, Chua & Ang 2016:1).

Majority of the respondents 57% (*n* = 56) were females, as compared with 43% (*n* = 43) males. This finding is similar to earlier studies (StatisticStats 2019:1; Tomas et al. 2019:114), which emphasised that nursing remain a female dominated profession despite more men joining the profession.

Regarding the religious orientation of the nurses, most of the nursing students 96% (*n* = 96) were Christians and only 4% (*n* = 3) represented other religions. This finding is a reflection of the dominance of Christianity amongst the nursing students at this campus. The findings also mirror the presence of Christianity in the Namibian population with 97% Christian and 3% non-Christian (U.S. Department of State 2020:1).

### Nursing students’ self-reported professional behaviour

Overall, the findings of our study indicated that self-reported professional behaviour of the nursing students was high with nursing students scoring relatively high mean scores (> 4.0) in all aspects of self-reported professional behaviour. These findings show a higher score of professional behaviour as compared with those reported in other studies; (2019a:1) mean score of 3.86 ± 0.17in a study by Poorchangizi et al. (2019a:1) and mean score of 3.91 ± 0.57 in a study by Mousaviasl et al. (2019:175). It is not clear why students in our study generally rated their professional behaviour high. Objective measurement of students’ professional behaviour is needed to validate these findings.

Despite high mean score in professional behaviour for age, gender and level of study, the study found no statistical significance difference between these demographic characteristics and professional behaviour.

The relationship between religion and self-reported professional competency was statistically significance (*p* = 0.01) as also between religion and quality care improvement (*p* = 0.02). These results should be interpreted with caution as the number of non-Christians in the study was low. All the same, the results reflect how nursing as a caring profession is rooted deeply in Christianity and other faith-related religions (Swihart, Yarrarapu & Martin 2020:3). In addition, religion is consistent with self-reported professional competency and quality improvement because altruism, honesty, integrity and human dignity are core values of both religion and the nursing profession, and its history (Poorchangizi et al. 2019b:1; Rieg, Newbanks & Sprunger 2018:3). Furthermore, Frondell and Pietilä Sarmiento (2015:7) helped to explain the link between religion and professional behaviour, stating that nurses are trained to practice with compassion, respect and individual value that are similar to the main teachings of Christianity.

Advocating for patients is a necessary skill for nurses and nursing students. In this study, nursing students had a high mean score of (4.09 ± 0.51). The results reflect a good nursing attribute that is recommended by American Nurses Association (2010:1), which states that nurses must always uphold the values and ethics of the profession by protecting the patients at all times. In support of the results, students were found to develop advocacy skills during their placements (Moquin, Seneviratne & Venturato 2018:10). However, there are limited studies evaluating the nursing students’ advocacy role with many studies focusing on professional nurses.

In this study, protecting health, safety and rights of the patients had a high mean score of (4.28 ± 0.55). This result is in line with an earlier study that reported that nursing students felt confident about their clinical safety skills (Huang et al. 2020:1). However, other studies reported contradictory results showing that nursing students had a low competence on patient safety (Noviyanti, Handiyani & Gayatri 2018:12). The differences in the results suggest that students tend to overestimate their competence in self-reported measurements only for objective measurements to show that their competence is not that high.

Utilisation of evidence in nursing practice is an important aspect of nursing practice. The results revealed that students rated their competence in utilisation of evidence-based highly with a mean of 4.78 ± 0.58. However, in a study on the implementation of evidenced practice, students reported low mean scores (Ashktorab et al. 2015:2). Similar results were reflected in a study amongst nurses who had low competence on evidence-based practice (Pashaeypoor et al. 2017:203). It is possible that students in this study lacked self-evaluation skills and overrated their evidenced based practice skills. It could also be that student nurses engage in research to solve daily patients’ health problems, although it is highly unlikely (Brower 2017:1).

Although, the overall professional behaviour scores were high, a low mean score (2.22 ± 1.27) on projecting a professional image was recorded. This confirms results from previous studies demonstrating how nurses fail to project their image to the public (Hoeve, Jansen & Roodbol 2013:306). If they manage to project their image, they lack confidence in doing so (Ndirangu et al. 2021:10). There is an urgent need for strategies at both school and professional level to help nursing students improve their public image and tell a story for nursing than to leave it in the hands of the public. Simultaneously, it is of importance for the nursing students and nurses to observe professional standards to project a good public image (Magopeni 2013:2).

Nursing students’ collaboration with other healthcare professionals and the community was rated high with a mean score of 4.16 ± 0.42. These results contradict existing literature where nursing students reported being insecure in collaborating with medical students, lacked confidence and understanding of team work (Lestari, Scherpbier & Stalmeijer 2020:1143; Oxelmark et al. 2017:14). Considering that nurses work as part of a team, there is need to help student develop collaboration skills (Oldland et al. 2020:151). There is evidence that some interventions can work to improve collaboration skills amongst healthcare students including nurses (Lestari et al. 2020:1143; Oxelmark et al. 2017:14).

### Implications for nursing education and practice

The nursing students rated their professional behaviour high compared with most findings, which reported low professional behaviour amongst nursing students and nurses. It is recommended to put more emphasis on objective assessemment of nursing students’ professional behaviour. Results of the present study can be useful for nurse educators to develop strategies for teaching professional behaviour. Furthermore, future studies should be directed on the role of mentors on how to improve existing students’ mentorship programmes. The role of religion on professionalism need to be further investigated using a larger sample.

### Strengths and limitations

Professionalism has proved difficult to teach and assess over the years (Ichikawa et al. 2020:1608; Li et al. 2017:2). The strength of this article lies in the assessment of professional behevaiour from the perespective of nursing students, contributing to literature on assessment of professionalism in nursing. This research illustarted the need to assess professional behevaiour amongst nursing both subjectively (self- reported) and objectively. Such an intervention can significantly contribute to the development of professional behaviour amongst nursing students. The study was conducted at one campus of the university with a relatively small sample size, therefore the generalisability of the findings is limited. Also, assessing the self-reported professional behaviour from student’s perspective alone is subjective and biased. There is a need for further investigation into the objective professional behaviour of students and also from the perspectives of nurse educators, clinical nurses and patients.

## Conclusion

In this study, nursing students’ self-reported professional behaviour was high (mean scores > 4.0 out of 5). This implies that nursing students believe that they are competent in professional behaviour and more likely to be confident in situations that demand professionalism (Lundberg 2008:1; Makarem et al. 2019:261). Despite the high self-reported behaviour, it cannot be concluded that the students were professionally competent. Some of their high ratings were in contrast to the established literature raising questions about the validity of their self-evaluation skills. Therefore, it is important that nursing education should facilitate the development of professional behaviour, measure it objectively and help students to build self-evaluation skills. The study found a low mean score on professional image, an area that needs to be addressed in order to promote this noble profession. The promotion of the nursing profession’s image should be in the hands of nursing students and nurses provided that they practice good professionalism.
